# Quality control of traditional Chinese medicine Chelidonii herba based on 2D-Q-NMR

**DOI:** 10.1016/j.heliyon.2024.e37405

**Published:** 2024-09-04

**Authors:** Huanbang Zhang, Mengjie Chen, Yang Xu, Kun Wang, Hua Li, Lixia Chen, Chao Huang

**Affiliations:** a Wuya College of Innovation, Key Laboratory of Structure-Based Drug Design & Discovery, Ministry of Education, Shenyang Pharmaceutical University, Shenyang, 110016, China; b Ningxia Institute for Drug Control, Ningxia, 750004, China; c Institute of Structural Pharmacology & TCM Chemical Biology, College of Pharmacy, Fujian University of Traditional Chinese Medicine, Fuzhou, 350122, China

**Keywords:** 2D-Q-NMR, Chelidonii herba, Quantification

## Abstract

Chelidonii herba is a traditional Chinese medicinal herb with effects including antispasmodic, analgesic, antitussive, and bronchodilator properties. Alkaloids are the main bioactive ingredients in Chelidonii herba. In this study, a two-dimensional nuclear magnetic resonance (Heteronuclear Singular Quantum Correlation, HSQC-2D-NMR) technique was employed to quantitatively analyze the total alkaloid content and three major active alkaloid monomers in Chelidonii herba from eleven different sources. The quantification results of the three monomeric alkaloids were also verified using conventional quantitative control methods such as HPLC. Experimental findings indicate that the total alkaloid content is not directly correlated with the content of the three monomeric alkaloids. Furthermore, the content of any individual monomeric alkaloid does not accurately reflect the overall quality of Chelidonii herba. It was demonstrated that the 2D-Q-NMR NMR can be applied as an alternative method. While maintaining the same levels of accuracy and precision, the 2D-Q-NMR method is simpler to operate and provides more comprehensive results with higher reproducibility in some cases.

## Highlights

• D-Q-NMR are used for the quantification of Chelidonii herba.
• D-Q-NMR demonstrates consistency with HPLC in terms of quantitative reliability.
• D-Q-NMR allows for the overall quantification of alkaloids.

## Graphical abstract

**Figure:**
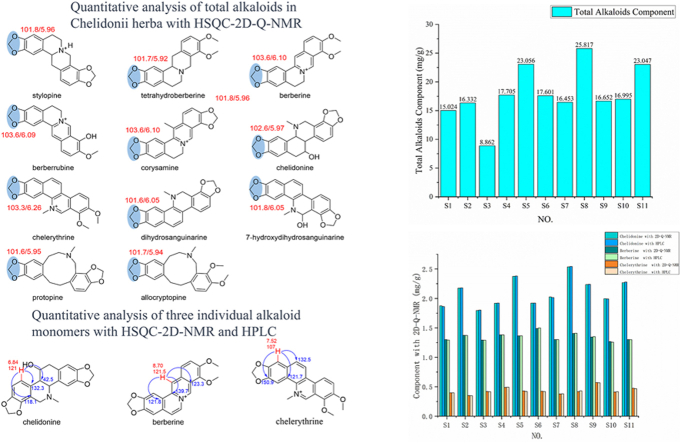

## 1 Introduction

Chelidonii herba as a traditional Chinese medicine, is the dried whole herb of the plant *Chelidonium majus* L. of the genus *Chelidonium* L. in the family Papaveraceae. It is widely distributed in regions such as Northeast China, Shandong, Anhui, Henan, Jiangsu, and Hubei. In traditional Chinese medicine theory, Chelidonii herba is believed to have the effects of antispasmodic, analgesic, antitussive, and bronchodilator. It is commonly used to treat conditions like gastric spasms, coughs, asthma, and pertussis. Modern pharmacological studies have revealed that Chelidonii herba exhibits various activities, including antibacterial [[1], [2], [3], [4], [5]], insecticidal [6,7], antiviral [8], antitumor [[9], [10], [11], [12], [13], [14], [15], [16], [17], [18], [19]], anti-inflammatory [20,21], analgesic [22,23], antispasmodic [24,25], anti-osteoporotic [26], and antioxidant [27] effects. The main components of Chelidonii herba are alkaloids, flavonoids, and phenolic acids, with alkaloids being the predominant and primary active compounds [28]. The three most significant alkaloid components are chelerythrine [1,2,[12], [13], [14], [15]], berberine [9,14,15] and chelidonine [3,13,[16], [17], [18],^27^].

Quantitative Nuclear Magnetic Resonance (Q-NMR) technology is a novel analytical technique based on the principles of nuclear magnetic resonance spectroscopy. This method offers several advantages, including accuracy, simplicity, high precision, good reproducibility, and non-destructiveness [29]. Q-NMR has found widespread applications in quantitative studies across various fields such as food [[30], [31], [32], [33]], pharmaceuticals [[34], [35], [36], [37], [38], [39], [40]], and the environment [41,42]. Q-NMR allows for the precise quantification of a compound's content based on its characteristic chemical shift values. It also allows for the simultaneous quantification of multiple different molecules in a single snapshot without the need for separation, derivatization, or purification. This method presents a significant advantage compared to traditional analyses.

Unlike High Performance Liquid Chromatography (HPLC), Q-NMR can also quantitatively determine the total content of a class of compounds sharing similar structural features and exhibit similar chemical shifts in NMR spectra [30,32]. This feature makes it particularly valuable for determining the most active compounds with a certain type of characteristic structure in complex chemical systems such as traditional Chinese medicine and natural products.

However, in complex systems with multiple components, 1D-Q-NMR may encounter issues with signal overlapping and shifting. In such cases, 2D-Q-NMR has been proposed as an optimized technique over 1D-Q-NMR. 2D-Q-NMR introduces an additional dimension, dispersing the overlapping signal peaks and yielding independent and reliable quantification points. This addresses challenges that 1D-Q-NMR cannot overcome [[33], [34], [35], [36], [37]]. When dealing with complex multi-component systems, 2D-Q-NMR offers greater advantages by enabling more accurate identification and quantification of various components. As a result, it is gradually gaining attention in the field of traditional Chinese medicine analysis. For example, in more than 40 known mogroside compounds from *Siraitia grosvenorii* (luo han guo), only 11-α-hydroxymogroside exhibits a sweet taste, while other triterpenoid compounds are tasteless or bitter. 2D-Q-NMR selectively quantifies the content of 11-α-hydroxymogroside in dried fruits, accurately predicting their sweetness potential [30]. In another wine system studied, carbonyl compounds are crucial oxidative products, but their prediction is challenging due to various complex associative reactions. 2D-Q-NMR can simultaneously and directly quantify five carbonyl compounds and their sulfite salts, hydrates, and carboxylic acid forms, comprehensively analyzing the occurrence and development of wine oxidation processes [32].

Currently, quantitative analysis of Chelidonii herba is mostly conducted using HPLC [[43], [44], [45], [46]]. Only J. Hua et al. [47] employed an acid dye colorimetric method to determine the total alkaloid content in Chelidonii herba, which marks the first study of quantifying total alkaloid content in this plant. Apart from these, thin-layer chromatography (TLC) is suitable only for qualitative analysis, and capillary electrophoresis [48] is limited to charged and polar compounds. Thus, a reliable, stable, and widely applicable quantitative analysis method for total alkaloid content in Chelidonii herba is currently lacking.

In this study, the 2D-Q-NMR technique was employed to identify characteristic structural points of total alkaloids and three individual alkaloids in Chelidonii herba. Quantitative analysis of the content of total alkaloids and three individual alkaloids in Chelidonii herba was conducted, aiming to provide a scientific basis for enhancing the quality standards of Chelidonii herba. Furthermore, this research lays the foundation for further pharmacological studies on Chelidonii herba.

## 2 Materials and methods

### 2.1 Instruments, reagents, and materials

Bruker AV 600 MHz spectrometer with a 5 mm TXI triple resonance probe, Bruker AV Ⅲ 400 MHz HD spectrometer in 298K, from Massachusetts, USA.

Waters 2695 HPLC System, Waters 2996 UV Full Wavelength Detector, XBridge Shield RP 18 chromatographic column(4.6 × 250 mm, 5 μm; Waters, Ireland), from Massachusetts, USA.

The deuterated solvent CD_3_OD used for NMR analysis was a product of Shanghai Haosu Chemical Technology Co., Ltd. Conventional 5 mm NMR sample tubes were obtained from Norell Inc. (Landisville, NJ, USA).

The 11 batches of Chelidonii herba samples (S1-S11) were collected from different regions, as shown in Table 1. The standard samples of chelidonine, berberine, and chelerythrine (>99.5 %, standards for HPLC) were purchased from Shanghai Yuanye Bio-Technology Co., Ltd.

**Table 1:** Summary of sample origin.

Sample	Origin	Sample	Origin
S1	Anhui, China	S7	Shandong, China
S2	Hebei, China	S8	Shanxi, China
S3	Henan, China	S9	Zhejiang, China
S4	Hubei, China	S10	Hunan, China
S5	Jiangsu, China	S11	Liaonig, China
S6	Jiangxi, China		

The source of the above-mentioned eleven batches of Chinese medicinal herbs has been authenticated by Wang Kun, the vice president of Ningxia Institute for Drug Control, confirming their origin to be genuine.

### 2.2 Sample preparation

#### 2.2.1 Samples for 2D-Q-NMR

2.0 g of herbal material from each of the 11 batches was weighed and placed in a conical flask. The flask was then filled with 100 mL of 80 % methanol solvent, and it was subjected to ultrasonic extraction at a frequency of 40 kHz for 2 h. After extraction, the solution was centrifuged, and all of the supernatant(about 15 mL) was collected. The solvent was evaporated, and then it was reconstituted with methanol to a final volume of 1.0 mL in a volumetric flask. 500 μL of deuterated methanol was accurately added to dissolve the residue and transfer it to a 5 mm NMR tube. This process results in the prepared sample solution for NMR.

#### 2.2.2 Sample for HPLC

2.0 g of herbal material from each of the 11 batches was weighed and placed in a conical flask. The flask was then filled with 100 mL of 80 % methanol solvent, and it was subjected to ultrasonic extraction at a frequency of 40 kHz for 2 h. After extraction, the solution was centrifuged, and all of the supernatant(about 15 mL) was collected. The solvent was evaporated, and then it was reconstituted with methanol to a final volume of 10 mL in a volumetric flask. This process results in the prepared sample solution for HPLC.

### 2.3 Preparation of standards

#### 2.3.1 Standards for 2D-Q-NMR

15 mg of chelidonine, berberine, and chelerythrine standard substances were weighed separately. They were then dissolved in deuterated methanol and transferred into a 5 mm NMR tube to obtain the standard solution (the total alkaloids were standardized using the berberine standard solution).

#### 2.3.2 Standards for HPLC

2 mg of the reference standard of chelidonine, 2 mg of berberine, and 1 mg of chelerythrine were weighed separately. They were accurately weighed and placed into 1 mL volumetric flasks. They were dissolved in methanol solvent and then made up to the mark to obtain the standard solution.

### 2.4 Analysis method

#### 2.4.1 Analysis method for 2D-Q-NMR

In the 2D-Q-NMR quantification experiment, the influence of scan numbers on the experiment was investigated. Scans were conducted with 4, 8 and 16 repetitions. The results showed that with 4 scans, the signal-to-noise ratio of the spectra was low; with 8 scans, the signal-to-noise ratio was higher and the spectra were more stable; with 16 scans, the signal-to-noise ratio was almost consistent with 8 scans. As increasing the number of scans would extend the experimental time, a scan number of 8 was ultimately chosen for the experiment.

For the final 2D-Q-NMR quantification experiment, a Bruker Avance 600 MHz nuclear magnetic resonance (NMR) spectrometer equipped with a 5 mm TXI triple resonance probe was utilized for detection. The experiments were conducted at a temperature of 298 K. The spectroscopic acquisition parameters were set as follows: the pulse sequence employed was the Bruker standard pulse sequence hsqcedetgpsp.3, the proton (^1^H) and carbon (^13^C) spectral widths were 10.01 ppm and 200 ppm, respectively. The ^1^H channel acquired 1024 data points, and the ^13^C channel acquired 256 data points. The experiment was repeated 8 times with a blank run of 16 times, and the acquisition time was 0.14 s. The relaxation delay time was set to 1.5 s. The total acquisition time for the 2D-Q-NMR quantification experiment was 55 min.

The nuclear magnetic resonance (NMR) spectra were processed using Bruker Topspin software version 2.1.0. The following data processing steps were applied to the raw data:

Zero filling: The data were zero-filled to achieve a 1k x 1k matrix size.

Multiplication with squared sinebell function: The two dimensions were multiplied using a squared sinebell function to improve the signal-to-noise ratio.

Fourier transformation and phase correction: The processed data underwent Fourier transformation and phase correction.

Chemical shift referencing: The solvent peak signal of deuterated methanol (*δ*_H_ 3.3 ppm; *δ*_C_ 49 ppm) was used as an internal reference to calibrate the chemical shifts.

Baseline correction: Manual baseline correction was performed on both carbon and hydrogen dimensions.

**Fig. 1:**
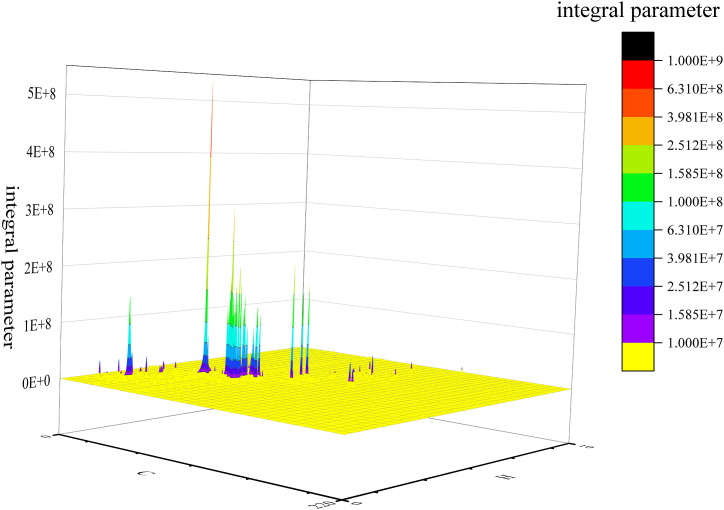
Signal interception height in the extract of Chelidonii herba.

Integration processing: After baseline correction, the same integration parameters were used for *δ*_C_: 0–200 ppm and *δ*_H_: 0–10 ppm ranges. The integral minimum threshold was set to the relative value of 0.02 (absolute value of 10830542.44) to remove signal background noise and impurities, roughly as shown in Fig. 1 above yellow.

This data processing workflow ensured accurate and consistent analysis of the NMR spectra for quantification and characterization purposes.

#### 2.4.2 Analysis method for HPLC

Column: XBridge Shield RP 18 column (4.6 × 250 mm, 5 μm; Waters, Ireland).

Mobile phase: A: Acetonitrile, B: 1 % triethylamine buffer (adjusted to pH 3.0 with phosphoric acid), gradient elution program as shown in Table 2.

**Table 2:** Gradient elution program.

t/min	ϕ(A)/%	ϕ(B)/%
0	10	90
5	15	85
55	20	80
70	25	75

Detection wavelength: 257 nm.

Flow rate: 1.0 mL/min.

Column temperature: 30 °C.

Injection volume: 20 μL.

#### 2.4.3 HPLC system suitability test

**Fig. 2:**
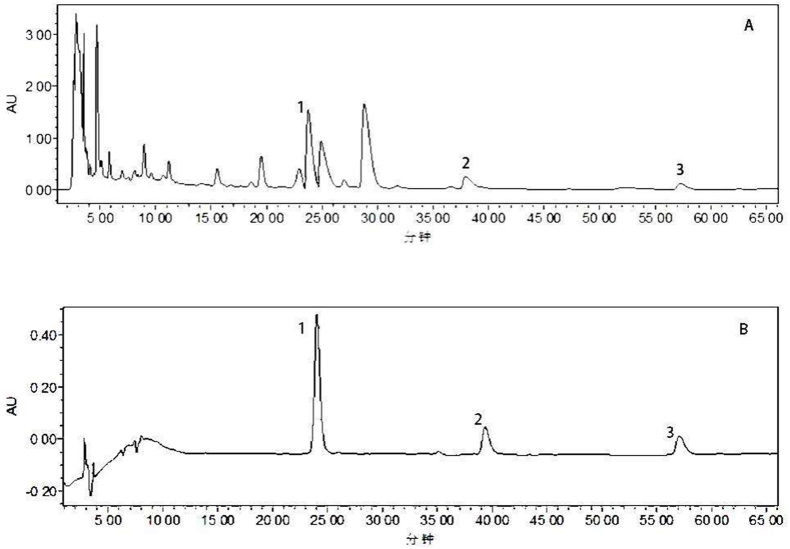
Sample solution (A); HPLC chromatograms of solution of mixed reference Standards (B) 1- chelidonine; 2-berberine; 3-chelerythrine.

The analysis was performed in accordance with the chromatographic conditions outlined in 2.2.2. The separation of the chromatographic peaks for the three components of interest is greater than 1.5, and the symmetry factors are within the required specifications. The results are presented in Fig. 2.

### 2.5 Method validation

The developed analytical method underwent rigorous validation to ensure its accuracy, precision, reliability, and robustness. The validation process encompassed several parameters, including linearity, precision, repeatability, accuracy and stability. The detailed summary of the validation results is provided below.

In the 2D-Q-NMR quantitative method, a good linear relationship was observed between the concentration of *Chelidonium* alkaloids within the range of 1–60 mg/mL and the integration value of the methylenedioxy signal, represented by the equation y = 7 × 10^10^x - 6 × 10^7^ with an R^2^ of 0.9991. Similarly, the linear relationships between concentration and characteristic quantification signal integration values were well-established for chelidonine and berberine, with equations y = 7 × 10^10^x + 5 × 10^6^ (R^2^ = 0.9981) for concentrations within 0.5–5.5 mg/mL and y = 4 × 10^11^x - 3 × 10^8^ (R^2^ = 0.9991) for concentrations within 0.5–5.5 mg/mL, respectively. For chelerythrine, within the range of 0.25–2.0 mg/mL, the relationship was represented by y = 6 × 10^11^x - 2 × 10^7^ (R^2^ = 0.9992).

In the HPLC quantitative method, chelidonine demonstrated a good linear relationship between concentration and the characteristic UV absorption peak area integration value within the range of 0.02–5.5 mg/mL, represented by y = 4 × 10^7^x + 36534 with an R^2^ of 0.9982. Similarly, for berberine within 0.02–5.5 mg/mL and chelerythrine within 0.01–2.0 mg/mL, the linear relationships were established with equations y = 7 × 10^7^x + 603216 (R^2^ = 0.9997) and y = 8 × 10^7^x + 126757 (R^2^ = 0.9991), respectively.

The precision of the instrument was evaluated by conducting 6 repeated measurements on the same standard solution. The *Chelidonium* alkaloids standard solution was subjected to 6 repeated measurements, resulting in an RSD value of 1.2 % for the integration value of the methylenedioxy signal in the *Chelidonium* alkaloids structures. Similarly, the standard solutions of chelidonine, berberine, and chelerythrine were each subjected to 6 repeated measurements using the 2D-Q-NMR and HPLC method. The RSD values for the integration values of the characteristic quantification points in 2D-Q-NMR were found to be 1.6 %, 1.0 %, and 0.38 % for chelidonine, berberine, and chelerythrine, respectively. Moreover, the RSD values for the quantification peak areas in HPLC were 1.4 %, 1.7 %, and 1.4 %. These results collectively demonstrated that the instruments exhibit good precision.

Repeatability analysis was conducted on six Chelidonii herba samples from Liaoning province, the RSD values for the quantitative points of total alkaloids using 2D-Q-NMR were 2.9 %. The RSD values for the characteristic quantitative points of the three individual alkaloids were 2.5 %, 0.70 %, and 1.1 % using 2D-Q-NMR, and 1.4 %, 1.7 %, and 1.4 % using HPLC. These results indicate good repeatability of the measurement results using this experimental method.

Stability testing involved measuring the quantification point integration values for total alkaloids, chelidonine, berberine, and chelerythrine in six Chelidonii herba samples from Liaoning province over 24 h, resulting in RSD values of 1.0 %, 1.0 %, 0.37 % and 1.6 % using 2D-Q-NMR. Chelidonine, berberine, and chelerythrine's RSD values for HPLC quantification peaks were 1.5 %, 1.1 %, and 1.6 %. This indicates stability of the sample solutions within the 24-h period.

Recovery assessment was performed by preparing nine parallel sample solutions of Chelidonii herba from Liaoning province, and after quantification by both 2D-Q-NMR and HPLC methods, corresponding standard solution concentrations (80 %, 100 %, 120 %) were added. The average recovery rate of total alkaloids in white aconitine using 2D-Q-NMR was 101.8 %, with an RSD of 2.2 %. The average recovery rates for chelidonine, berberine, and chelerythrine were 101.8 %, 102.6 % and 101.1 %, respectively, using 2D-Q-NMR, with RSD values of 2.2 %, 0.82 %, and 1.3 %. The average recovery rates for HPLC were 96.8 %, 95.3 %, and 103.3 %, with RSD values of 2.5 %, 2.1 %, and 2.1 %.

The methodological validation demonstrates that the developed analytical method is accurate, precise, reliable, and durable for quantifying *Chelidonium* alkaloids in various samples. The results indicate that these methods are fit for their intended purpose and provide consistent and accurate quantitative data.

## 3 Results

### 3.1 Quantification of total alkaloids with 2D-Q-NMR

**Fig. 3:**
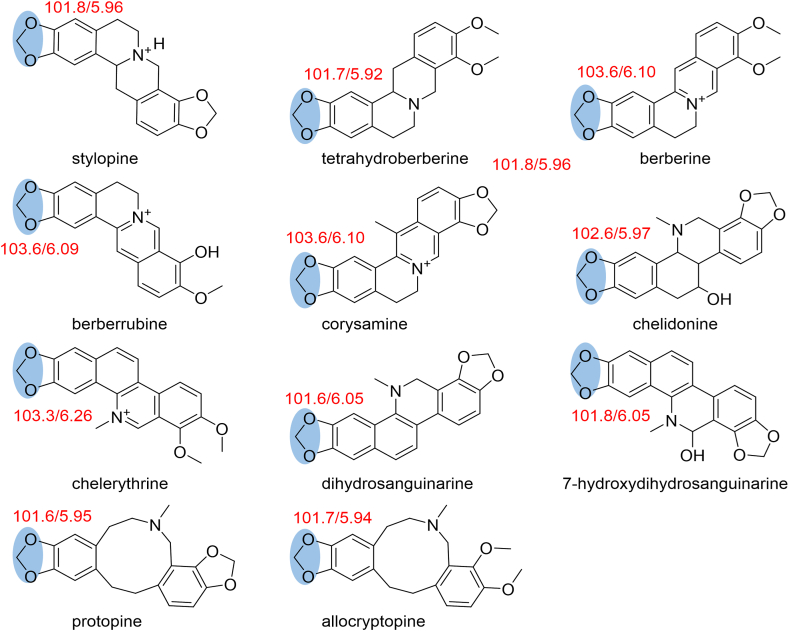
Common structural characteristic signal point of 11 alkaloids in Chelidonii herba.

**Fig. 4:**
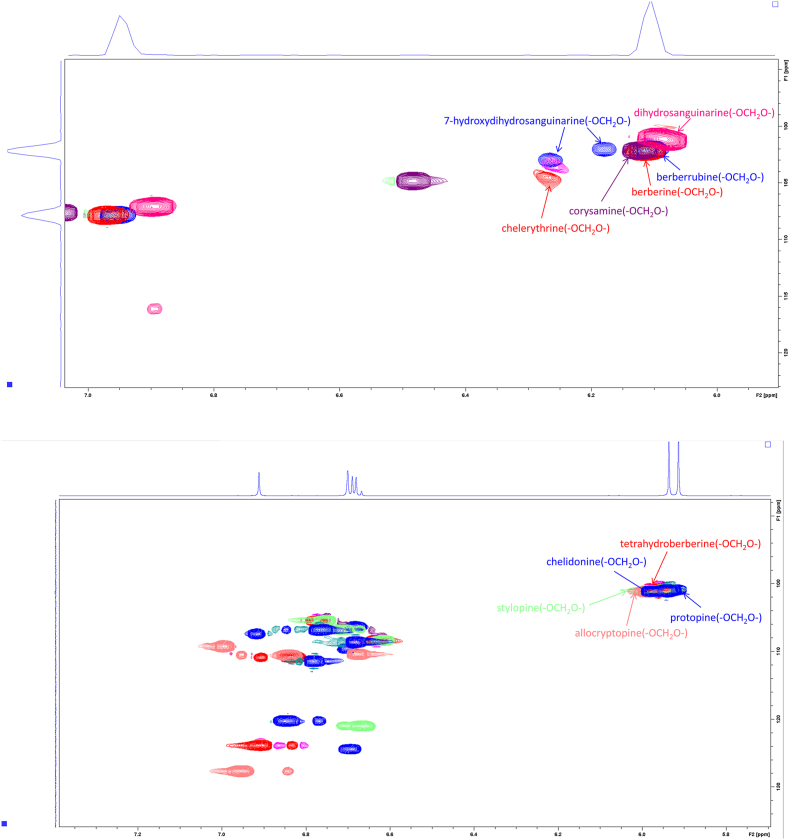
Comparison of the methylenedioxy signal peaks among the 11 alkaloid standard compounds.

**Fig. 5:**
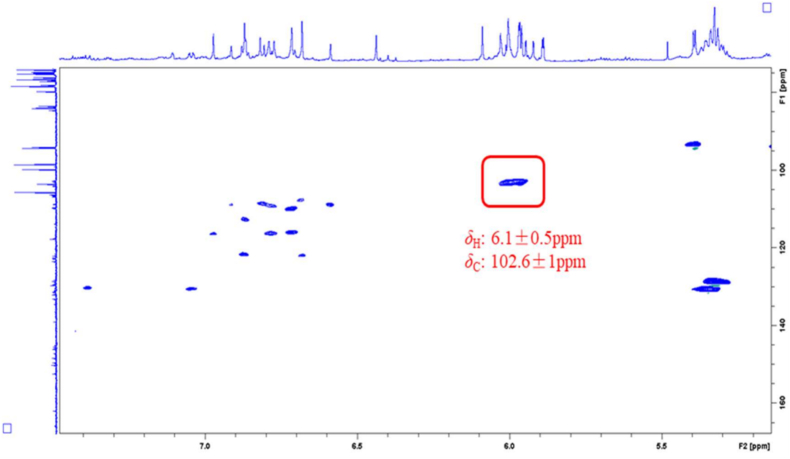
Key HSQC correlations of total alkaloids with a methylene dioxy structure in the extract of Chelidonii herba.

The attribution of qHSQC signals corresponding to the common structural motif (methylenedioxy) of the 11 alkaloid standards selected based on their relative content and activity in Chelidonii herba is summarized in Fig. 3. Meanwhile, a comparative analysis was conducted by comparing the qHSQC spectra of the 11 alkaloid standards (Fig. 4) with the qHSQC spectrum of the crude extract of Chelidonii herba (Fig. 5). It was observed that the resonance signal peaks of the methylenedioxy group appeared at *δ*_H_ 6.1 ± 0.5 ppm and *δ*_C_ 102.6 ± 1 ppm positions for all 11 alkaloids, without any other interfering signals. Hence, the methylenedioxy signal peak can be chosen for quantifying the content of total alkaloids.

**Fig. 6:**
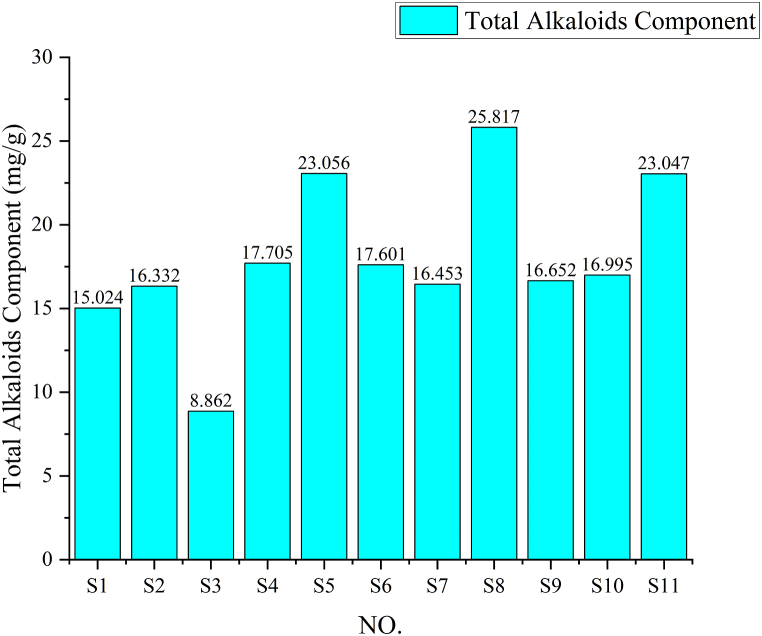
Contents of total alkaloids in Chelidonii herba.

Precisely weighed 2.0 g of each of the 11 batches of Chelidonii herba samples were taken. The test samples were prepared according to the method described under "2.2.1". The determinations were conducted under the experimental conditions outlined in "2.3.1″, and the peak area integration values of the quantification signal peak for total alkaloids in Chelidonii herba were recorded. Subsequently, the content of total alkaloids was calculated using the standard curve method. The results are presented in Fig. 6.

From Fig. 6, it can be inferred that the batch S8 from Shanxi has the highest total alkaloid content among all batches. The batch S5 from Jiangsu and S11 from Liaoning follow next in terms of content. The batch S3 from Henan has the lowest content, being only one-third of S8's content. The alkaloid content in the remaining regions is approximately two-thirds of that in S8.

### 3.2 Quantification of three individual alkaloids with 2D-Q-NMR

**Fig. 7:**
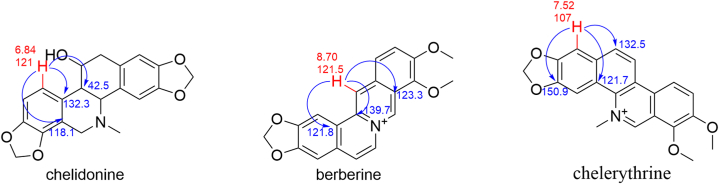
Quantitative signal point of chelidonine, berberine and chelerythrine.

**Fig. 8:**
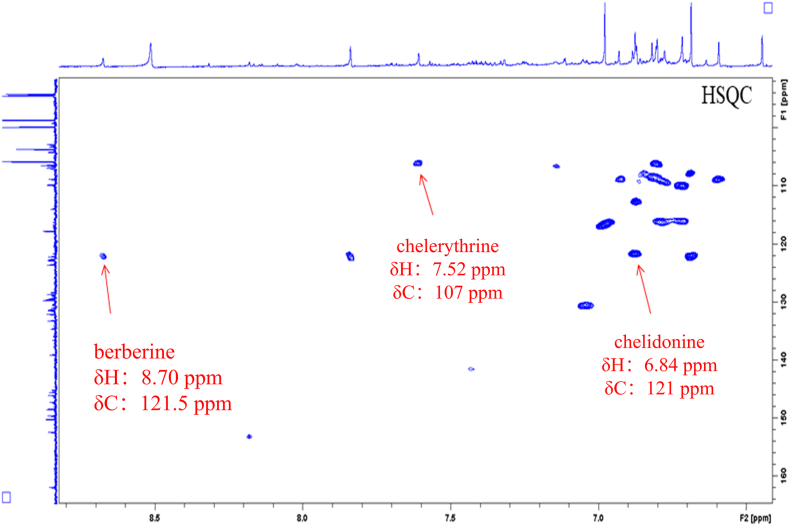
Key HSQC correlations of chelidonine, berberine and chelerythrine in the extract of Chelidonii herba.

**Fig. 9:**
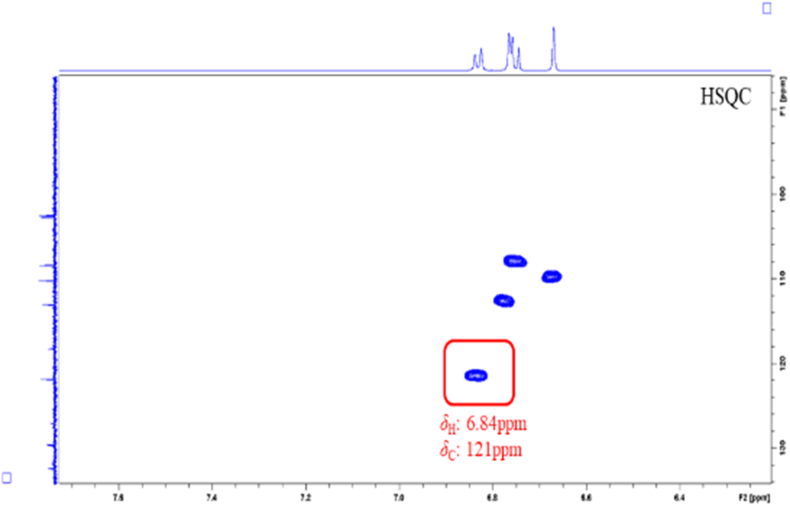
Key HSQC correlations of chelidonine standard.

**Fig. 10:**
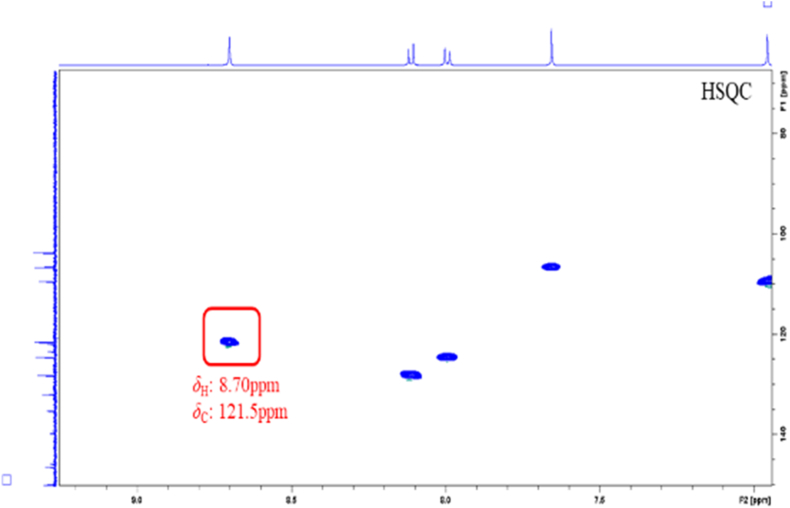
Key HSQC correlations of berberine standard.

**Fig. 11:**
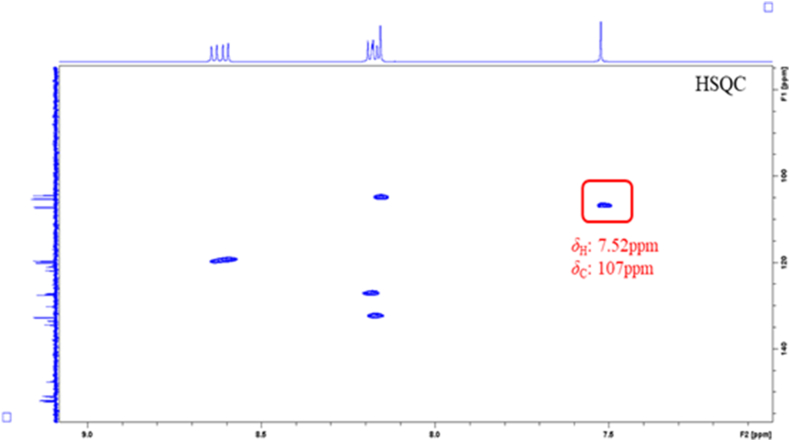
Key HSQC correlations of chelerythrine standard.

By comparing the attributions of HSQC signals of the alkaloids obtained from the Chelidonii herba (as shown in Table 3), it can be determined that by the 2D-Q-NMR spectrum of Chelidonii herba crude extract (Fig. 8), including the *δ*_H_ 6.84 (H-11)/*δ*_C_ 121 (C-11) HSQC cross-peak signal generated by chelidonine, the *δ*_H_ 8.70 (H-12)/*δ*_C_ 121.5 (C-12) HSQC cross-peak signal generated by berberine, and the *δ*_H_ 7.52 (H-1)/*δ*_C_ 107 (C-1) HSQC cross-peak signal generated by chelerythrine. These signal does not overlap with other peaks and exhibit good separation, which can be utilized for quantifying the content of chelidonine, berberine and chelerythrine. The quantitative points of the three compounds are shown in Fig. 7. Moreover, through the comparison of the HSQC spectra of Chelidonii herba alkaloids with the standards of chelidonine, berberine and chelerythrine (Fig. 9, Fig. 10, Fig. 11), the accuracy of the quantification signal points in the Chelidonii herba crude extract has been further confirmed.

**Table 3:** HSQC Signal comparison of 11 alkaloids.

position	HSQC (*δ*_C_/*δ*_H_ ppm)										
	stylopine	tetrahydroberberine	berberine	berberrubine	corysamine	chelidonine	chelerythrine	dihydrosanguinarine	7-hydroxydihydro sanguinarine	protopine	allocryptopine
1	105.6/6.73	105.5/6.73	106.5/7.66	106.4/8.59	109.2/7.02	110.0/6.67	107/7.52	104.3/7.10	104.6/7.69	106.8/6.64	109.2/6.95
2	145.9	145.8	149.9	149.9	149.2	149.7	151.7	148.1	147.3	146.0	148
3	146.1	146.1	152.0	151.9	151.2	147	150.9	147.4	148.0	148.0	147.3
4	108.3/6.59	108.3/6.59	109.3/6.96	109.3/6.94	111.8/7.33	112.9/6.78	105/8.15	100.7/7.66	100.5/7.12	108.0/6.90	110.5/6.63
4a	127.6	127.6	131.8	131.6	121.9	126.9	121.7	126.5	126.8	135.6	132.8
5	29.3	29.5	28.1	28.3	29.0	63.9/3.66	133.3	142.5	138.1	31.3	32.3
6	51.1	51.3	57.1	56.9	58.5	–	–	–	–	57.6	57.5
7	52.8	53.9	146.4/9.76	145.9/9.77	143.8/9.72	54.3	152/9.95	48.4/4.19	85.7/5.37	51.1/3.60	50.1
7a	116.5	127.8	123.3	121.1	134.7	118.1	119.8	113.6	113.1	117.3	128.5
8	143.2	150.2	145.7	147	148.5	144.4	147.4	144.6	145.2	146.2	151.5
9	144.9	145	152.1	146.6	146.6	146.9	150.7	147	147.2	146	146
10	106.7/6.69	110.9/6.79	128.0/8.12	119.3/7.72	112.8/7.90	108.3/6.76	127.3/8.19	107.1/6.85	108.8/6.94	110.3/6.69	110.4/6.80
11	121/6.64	123.8/6.87	124.5/8.00	126.3/8.01	120.6/8.00	121/6.84	120.8/8.64	116.1/7.30	116.3/7.77	124.9/6.66	127.7/6.91
11a	128.3	128.6	135.1	134.3	134.4	132.3	129.9	127.2	125.7	128.6	129.5
12	36.2	36.4	121.5/8.70	119.2/8.59	132.7	42.5/3.06	127.0	124.3	122.7	45.9	46.2
12a	59.7/3.59	60.1/3.54	139.7	138.6	137.4	–	–	–	–	–	
13	130.4	130.8	121.8	122.1	121.3	74.1/4.20	119.4/8.60	120.3/7.69	120.1/7.42	194.2	193.3
14	–	–	–	–	–	39.9	132.5/8.16	123.9/7.48	123.7/7.49	132.2	136
14a	–	–	–	–	–	129.5	134.2	130.7	131.0	–	–
-OCH_2_O-	101.8/5.96	101.7/5.92	103.6/6.10	103.6/6.09	103.6/6.10	102.6/5.97	103.3/6.26	101.6/6.05	101.8/6.05	101.6/5.95	101.7/5.94

Each of the 11 batches of Chelidonii herba samples was precisely weighed with 2.0 g. The sample solutions were prepared according to the method described under section "2.2.1". The quantitative experiments were conducted under the conditions specified in section "2.3.1″, and the peak area integration values of the quantification signals for chelidonine, berberine, and chelerythrine were recorded. Subsequently, the content of these three components was calculated using the standard curve method. The results are presented in Table 4.

**Table 4:** Contents of chelidonine, berberine and chelerythrine in Chelidonii herba by 2D-Q-NMR.

No.		Component (mg/g)	
	chelidonine	berberine	chelerythrine
S1	1.875	1.301	0.397
S2	2.175	1.374	0.351
S3	1.794	1.294	0.420
S4	1.918	1.381	0.489
S5	2.376	1.365	0.429
S6	1.921	1.490	0.423
S7	2.027	1.302	0.378
S8	2.537	1.405	0.419
S9	2.237	1.345	0.572
S10	1.996	1.267	0.409
S11	2.267	1.302	0.476

Among the eleven batches of Chelidonii herba samples, the batch S8 exhibited the highest chelidonine content, followed by batches S5, S9, S11, and S2, while the remaining batches had similar content. In terms of berberine content, batch S6 had the highest content, followed by batches S8, S4, S5, S9, and S2, while the remaining batches had similar content. Regarding chelerythrine content, batch S9 had the highest content, followed by batches S4 and S11. Batches S2 and S7 had the lowest content, while the content of the other batches was relatively similar.

### 3.3 Quantification of three individual alkaloids with HPLC

2.0 g of 11 batches of Chelidonii herba samples from different origins were precisely weighed. The samples were prepared into test solutions following the method described in "2.2.2". Then, the analysis should be carried out according to the experimental conditions outlined in '5.4.2,' and the peak areas of chelidonine, berberine, and chelidonine should be recorded. The content of these three components should be calculated using the standard curve method. The results will be presented in Table 5.

**Table 5:** Contents of chelidonine, berberine and chelerythrine in Chelidonii herba by HPLC.

No.		Component (mg/g)	
	chelidonine	berberine	chelerythrine
S1	1.862	1.294	0.397
S2	2.180	1.372	0.475
S3	1.803	1.291	0.351
S4	1.922	1.381	0.423
S5	2.380	1.365	0.420
S6	1.922	1.499	0.409
S7	2.016	1.301	0.419
S8	2.542	1.409	0.573
S9	2.240	1.351	0.429
S10	1.992	1.257	0.377
S11	2.278	1.300	0.489

**Fig. 12:**
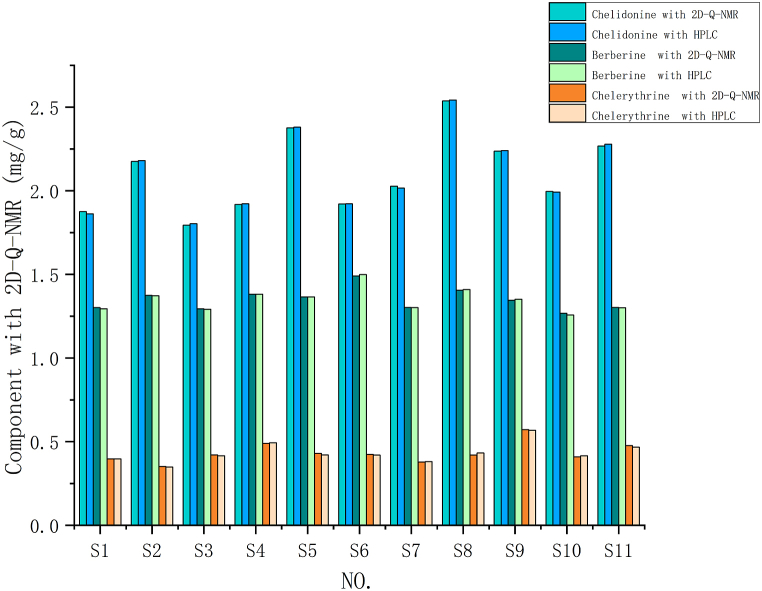
Comparison between the quantitative content determined by 2D-Q-NMR and HPLC.

The comparison between the quantitative content determined by 2D-Q-NMR and HPLC as shown in Fig. 12. Paired t-tests were conducted to compare the data obtained from 2D-Q-NMR. The results showed that for chelidonine, berberine, and chelerythrine, a p > 0.1 coefficient was found. Therefore, it can be concluded that there is no significant difference among them.

## 4 Discussion

The experimental results from this study indicate that the current method for quantifying the content of chelerythrine in Chelidonii herba using HPLC may not completely reflect the total content of alkaloids, the effective component in Chelidonii herba. For example, in batch S5 from Jiangsu province, the content of chelerythrine is not high, but its total alkaloid content ranks second among the eleven samples, and batch S6 from Jiangxi province, although the contents of total alkaloids, chelidonine, and chelerythrine contents are not remarkable, it has the highest content of berberine. Particularly noteworthy is in batch S3 from Henan province, the content of all three individual alkaloids isn't significantly lower than other batches, but its total alkaloid content is 2–3 times lower than that of other batches. Overall, batches S8 from Shanxi province, S11 from Liaoning province, and S5 from Jiangsu province have relatively higher contents of total alkaloids and individual alkaloid. This provides valuable reference for quality control and practical application of Chelidonii herba. This also suggests that the current method of using single standard substances for quality control of complex composition in herbal medicines exist limitations.

In terms of quantitative techniques, 2D-Q-NMR, as an advanced technology that integrates nuclear magnetic resonance with quantitative analysis, holds tremendous potential in the field of quality control of herbal medicine. In the quantification of major components in Chelidonii herba, its key advantages include three aspects. First, simplified sample preparation: Unlike HPLC, there is no need for trial-and-error exploration of separation conditions, which can significantly reduce the workload for establishing large-scale multi-index quantification systems for herb medicines. Second, high precision and excellent reproducibility: NMR spectra are not influenced by various factors of HPLC, such as mobile phase ratios, column types and conditions, elution programs, column pressures, and temperatures. With the proper usage of deuterated reagents, correct quantification procedures, and well-maintained instruments, highly consistent reproducible spectra can be easily obtained. Third, multicomponent quantification: 2D-Q-NMR can simultaneously detect the total content of specific types of compounds and multiple compounds, enabling multi-dimensional quantification of complex herbal medicine samples. These advantages show that 2D-Q-NMR has the potential to revolutionize the quality control of herbal medicines, offering reliable and accurate results for component analysis and quality evaluation.

It is essential to recognize that 2D-Q-NMR also has its shortcomings in certain aspects. The most notable is the significant difference in LOD (Limit of Detection) and LOQ (Limit of Quantitation) compared to HPLC. Taking this study as an example, the LOD and LOQ for 2D-Q-NMR can be determined at the 0.x mg level, whereas conventional HPLC can achieve LOD and LOQ at the μg level, indicating an order of magnitude difference. Currently, 2D-Q-NMR is still suitable for quantitative analysis at the mg level. Secondly, the high purchase price of NMR instruments and the equally high cost of subsequent maintenance limit the widespread adoption of this technology. Although in China, for instance, the number of NMR installations has increased in recent years, it is important to see that the installation and maintenance costs have not significantly decreased, and it is also difficult to foresee them reaching the level of popularity of HPLC in the foreseeable future. Fortunately, once linear calibration is performed, the quantitative results of Q-NMR on different instruments show almost no difference. It is not necessary, as with HPLC, to conduct a system suitability test before quantification. This makes it a viable option for third-party testing institutions, especially for organizations that do not possess an NMR spectrometer but wish to conduct Q-NMR research, and it can yield convincing and consistent results. Lastly, the cost of deuterated reagents, which is higher compared to that of chromatographic purity reagents, is also considered one of the barriers to scaled application. However, when considering the amount of mobile phase required for the entire process of chromatographic system checks, column equilibration, and injection, the use of 500 μL of deuterated reagent per injection may not necessarily be significantly higher than the former.

Of course, beyond the aforementioned issues, research on 2D-Q-NMR is not yet widespread, and the specific application scenarios and scope still require further clarification. It is foreseeable that not all traditional Chinese medicinal herbs or complex systems will have sufficient independent quantification points for systematic quantitative research. Additionally, not all similar types of compounds will have structurally identical overlapping signals for overall quantification. In such cases, 2D-Q-NMR cannot achieve quantitative purposes like HPLC by adjusting separation conditions. It may be necessary to consider using pre-treatment methods such as HPLC, TLC, etc., to appropriately process the samples before conducting quantitative research.

## 5 Conclusion

The 2D-Q-NMR technique accurately determined the content of total alkaloids, as well as the individual alkaloids including chelidonine, berberine, and chelerythrine, in 11 batches of samples. The methodological validation of 2D-Q-NMR exhibits good precision and accuracy. Moreover, there was no significant difference between the quantification results of the individual alkaloids obtained using 2D-Q-NMR and those obtained using HPLC, indicating that the 2D-Q-NMR technique is a reliable method for quantifying these alkaloids in Chelidonii herba.

## Ethics approval and consent to participate

Not applicable.

## Statement of originality

The authors declare that this manuscript is original, has not been published before, and is not currently being considered for publication elsewhere. We confirm that all sources of data and ideas presented in this work, which are not our own, have been appropriately cited and referenced in accordance with academic standards. The work presents original research not previously submitted or published, and all authors have read and approved the manuscript.

## Data availability

Data will be made available on request.

## CRediT authorship contribution statement

**Huanbang Zhang:** Writing – original draft, Visualization, Investigation, Data curation. **Mengjie Chen:** Resources, Methodology, Investigation, Formal analysis. **Yang Xu:** Writing – review & editing, Supervision. **Kun Wang:** Validation, Resources. **Hua Li:** Writing – review & editing, Supervision, Project administration. **Lixia Chen:** Writing – review & editing, Validation, Supervision. **Chao Huang:** Writing – review & editing, Supervision, Funding acquisition, Conceptualization.

## Declaration of generative AI and AI-assisted technologies in the writing process

During the preparation of this work the author(s) used ChatGPT 3.5 in order to improve grammar and enhance readability. After using this tool/service, the author(s) reviewed and edited the content as needed and take(s) full responsibility for the content of the publication.

## Declaration of competing interest

The authors declare that they have no known competing financial interests or personal relationships that could have appeared to influence the work reported in this paper.
